# Extracellular Vesicles from 50,000 Generation Clones of the *Escherichia coli* Long-Term Evolution Experiment

**DOI:** 10.3390/ijms232314580

**Published:** 2022-11-23

**Authors:** David Laurin, Corinne Mercier, Nyamekye Quansah, Julie Suzanne Robert, Yves Usson, Dominique Schneider, Thomas Hindré, Béatrice Schaack

**Affiliations:** 1 Département Scientifique Auvergne Rhône-Alpes, Etablissement Français du Sang, 38000 Grenoble, France; 2 Institute for Advanced Biosciences, INSERM U1209 & CNRS UMR 5309, Université Grenoble Alpes, 38042 Grenoble, France; 3 CNRS, UMR 5525, VetAgro Sup, Grenoble INP, TIMC, Université Grenoble Alpes, 38000 Grenoble, France; 4 CEA, CNRS, IBS, Université Grenoble Alpes, 38044 Grenoble, France

**Keywords:** extracellular vesicles (EVs), outer membrane vesicles (OMVs), Long-Term Evolution Experiment (LTEE), membrane fusion, LipoPolySaccharide (LPS), OmpA

## Abstract

Extracellular vesicles (EVs) are critical elements of cell–cell communication. Here, we characterized the outer membrane vesicles (OMVs) released by specific clones of *Escherichia coli* isolated from the Long-Term Evolution Experiment after 50,000 generations (50K) of adaptation to glucose minimal medium. Compared with their ancestor, the evolved clones produce small OMVs but also larger ones which display variable amounts of both OmpA and LPS. Tracking ancestral, fluorescently labelled OMVs revealed that they fuse with both ancestral- and 50K-evolved cells, albeit in different proportions. We quantified that less than 2% of the cells from one 50K-evolved clone acquired the fluorescence delivered by OMVs from the ancestral strain but that one cell concomitantly fuses with several OMVs. Globally, our results showed that OMV production in *E. coli* is a phenotype that varies along bacterial evolution and question the contribution of OMVs-mediated interactions in bacterial adaptation.

## 1. Introduction

All types of cells from bacteria, fungi, plants and animals produce extracellular vesicles (EVs) [1,2,3]. The diameter of these vesicles ranges from 30 to 1000 nm and their composition depends on both the type of cell they originate from and their mechanism of biogenesis. The main known role of EVs is to facilitate intercellular communication through the transport of molecules such as lipids, nucleic acids, proteins, sugars and metabolites [4,5,6]. EVs constitute a universal mechanism for inter-kingdom and intra-kingdom communication that is preserved among prokaryotic and eukaryotic cells [7,8].

Outer Membrane Vesicles (OMVs) constitute a particular sub-type of EVs produced by Gram-negative bacteria. They are natural proteo-liposomes containing soluble proteins and nucleic acids. OMVs are surrounded by one double leaflet membrane containing phospholipids, glycolipids, lipopolysaccharides (LPS) and membrane proteins [5,9]. OMVs follow different formation routes, which can lead to distinct OMVs subtypes with different molecular cargo and thus potentially different biological functions [4,10]. Two main processes have been proposed to trigger OMVs production, namely outer membrane blebbing and explosive cell lysis [4]. In the former case, structural changes in the cell envelope cause outer-membrane budding and the liberation of OMVs independently of cell lysis. In the second case, active degradation of the cell wall causes whole-cell lysis and the release of OMVs surrounded by a unique lipid bilayer as well as outer–inner membrane vesicles (O-IMVs) that are vesicles surrounded by two membranes. Based on this difference in biogenesis, it has been proposed that EVs resulting from explosive cell lysis are more prone to contain intracellular compounds including nucleic acids as compared to outer-membrane blebbing-derived OMVs, which mainly encapsulate soluble periplasmic content [4,5]. The production of OMVs with various contents is considered as a directed and selective cellular process with associated physiological roles as diverse as cellular communication, horizontal gene transfer, interbacterial killing, nutrient hydrolysis and stress responses [9]. However, the determinant of OMV biogenesis, the mechanisms responsible for OMV cargo selection as well as the contribution of these intercellular communication vectors to the adaptation of bacterial populations are not yet fully understood. Whether a given bacterial species systematically produces the same type of OMVs is still an open question. If not, what are the genetic changes susceptible to modulate both the biogenesis and the content of OMVs? Do OMVs constitute intercellular vectors whose content contributes to the evolutionary fate of bacterial populations?

We started to tackle these questions by investigating the dynamics of OMVs production in *Escherichia coli* populations from the Long-Term Evolution Experiment (LTEE) for which both phenotypic and genetic adaptations have already been thoroughly characterized [11]. In 1988, 12 independent populations were founded from two variants of the same *E. coli* B strain. These variants differed only by their capacity to catabolize arabinose. Six populations were initiated by the REL606 (Ara^–^) strain and six others by the near-identical REL607 (Ara^+^) strain. Since then, these 12 populations—called Ara^–1^ to Ara^–6^ and Ara^+1^ to Ara^+6^, respectively—have been propagated daily in 10 mL of glucose-limited minimal medium by transferring 1% of the previous day’s culture into an equal volume of fresh medium. This daily 100-fold regrowth corresponds to about 7 bacterial generations so that these 12 populations have now been propagated for more than 76,000 generations [12]. Every 500 generations, a sample of each bacterial population is cryopreserved, providing us with a frozen but viable bacterial fossil record that can be revived at any time for later analysis. The 12 populations have adapted by natural selection to the LTEE conditions with several parallel phenotypic changes [11,13]. First, the fitness of all 12 populations relative to their ancestor continuously increases over time at a diminishing rate consistent with a power law function predicting that fitness may continue to increase indefinitely [14,15]. Second, these fitness changes correlate with the increase in mean cell volume over time in all 12 populations, eventually in association with cell-shape changes making evolved cells more spherical than ancestral ones [11,16]. In two cases, the mutations causing such cell morphology changes were identified in genes related to peptidoglycan biosynthesis, and demonstrated as beneficial in the LTEE conditions [17,18]. Third, a majority of the 12 populations evolved a hyper-mutator phenotype [19] either due to DNA repair defects causing a ~100-fold increase of the point mutation rate [20] or to enhanced intragenomic mobility of Insertion Sequence [21]. Besides these parallel evolutionary changes, some LTEE populations exhibit unique phenotypic innovations. This is the case in the Ara^−2^ population where two lineages diverged from their common ancestor early in the experiment and established a long-term coexistence based on ecological niche construction and cross-feeding interactions [22,23].

In this study, we compared the biophysical and biochemical properties of OMVs produced by the LTEE ancestral strain versus 4 evolved clones isolated at 50,000 generations (50K) from populations exhibiting adaptive traits representative of those previously characterized in the 12 populations of the LTEE. The first 50K-evolved clone is from the hypermutator Ara^–1^ population [21] and harbors an adaptive mutation affecting one peptidoglycan-precursor biosynthesis gene [19]; the second and third clones are from the Ara^–2^ population. Each of these two clones belongs, respectively, to one of the two ecotypes that established long-term co-existence in this population owing to negative frequency-dependent and cross-feeding interactions [23]. The fourth clone is from the non-hypermutator Ara^–5^ population and possesses an adaptive mutation that affects peptidoglycan assembly [18]. We found that all four selected LTEE 50K-evolved clones similarly differ from their common ancestral strain for the size and the number of vesicles they produce per bacterial cell. Apart from these parallel changes most likely resulting from natural selection in the LTEE conditions, we also demonstrated that the evolved clones differ in both the protein and the LPS contents of their respective OMVs, hence suggesting that each evolved clone possesses genetic peculiarities, which lead to specific modulation of the biogenesis and the content of their respective OMVs. Finally, we showed the propensity of these OMVs to fuse with bacterial cells, hence questioning the role of their content in bacterial adaptation to new environmental conditions, including in the context of host–bacteria interactions (see companion paper for the propensity of these OMVs to fuse with human blood cells).

## 2. Results

### 2.1. OMVs Isolation from Pure Cultures of LTEE Strains

**Figure 1:**
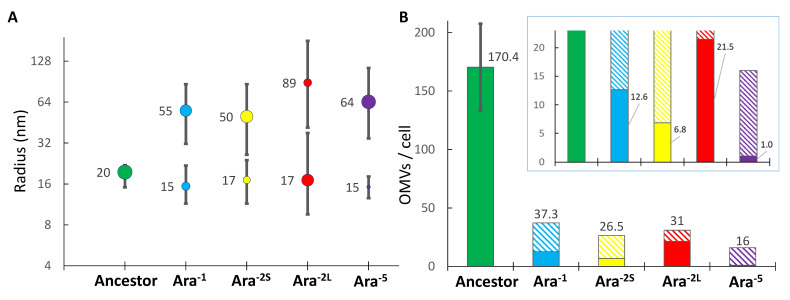
*E. coli* adaptation to the LTEE conditions causes biophysical changes in OMV production. OMVs were recovered from 1 L culture in DM1000 harvested at mid-log phase of growth for 5 selected clones: the LTEE ancestral strain (REL606) and four 50K-evolved clones isolated from the populations Ara^–1^ (REL11330), Ara^–2^ (S lineage, REL11335; L lineage, REL11333) and Ara^–5^ (REL11339). (**A**) Weighted average radii of OMVs as determined using Dynamic Light Scattering. Colored dots and associated values indicate the weighted average radius for one or two OMV size ranges for which both the lower and the upper bounds are indicated by the vertical bars. The surface of each dot is proportional to the OMVs’ abundance in each size range. (**B**) Number of OMVs produced per cell calculated as indicated in the material and methods section and detailed in Figure S1C. Non-hatched and hatched histograms correspond to small and large OMVs, respectively. The total number of OMVs produced per bacterial cell is indicated above each histogram. The inset panel corresponds to the same data but re-scaled to allow visualization of the number of small OMVs produced per cell by each evolved clone. For the ancestral strain (Ancestor), the value corresponds to the mean of 5 replicates (error bar shows the standard deviation). For the evolved clones, the value is from one experiment.

The culture medium used for propagating LTEE populations was Davis minimal medium (DM) containing glucose as the unique carbon source for *E. coli* and at low concentration (25 mg/L) causing bacterial growth to stop when cell density reaches about 5.10^7^ cell/mL. Because we anticipated that these culture conditions would prevent isolation of a sufficient number of OMVs for further characterization, we decided to quantify OMVs production by the *E. coli* B ancestor in Davis medium supplemented with 40 times more glucose to increase cell density. The REL606 ancestral strain was grown in 1 L of DM1000 (1000 mg/L of glucose) until the culture reached the mid-log phase. OMVs from this pure culture were recovered and first analyzed using Dynamic Light Scattering to determine the size distribution of isolated OMVs (Figure S1A). The radii of OMVs from the ancestral strain were rather homogeneous, ranging from 15 to 26 nm with a weighted average of 19.6 nm (Figure 1A and Figure S1A). Having calculated the total lipid content present in the OMV preparation, we found (see material and methods section and Figure S1) it to correspond to 4.8 (±0.96) × 10^13^ OMVs or 170 ± 37 OMVs per bacterial cell (Figure S1C). Because our procedure allowed the recovery of a sufficient amount of OMVs for further characterization, bacterial cultures in DM1000 up to mid-log phase were used for all the experiments reported thereafter.

### 2.2. OMVs Biophysical Features Have Changed over 50,000 Generations of E. coli Evolution

We repeated the exact same procedure but using pure cultures of each of the four selected LTEE 50K-evolved clones, namely Ara^–1^, REL11330; Ara^–2^, S lineage, REL11335; Ara^–2^, L lineage, REL11333; Ara^–5^, REL11339. Contrary to the ancestral strain, all 50K-evolved clones produced two types of OMVs, one with a low radius ranging from 15 to 17 nm (small OMVs), and the other with a larger radius comprised between 55 and 89 nm (large OMVs) (Figure 1A). The OMVs’ size distribution for all evolved clones also differed from that measured for the ancestor with both small and large OMVs encompassing wider size ranges (Figure 1A and Figure S1A). Moreover, comparing the size distribution of OMVs between evolved clones revealed that the proportion of small and large OMVs they produced differed. Small OMVs constituted 34% of the total number of OMVs produced by the Ara^–1^ clone and as few as 6% for the Ara^–5^ clone. Strikingly, the clones from each of the two ecotypes that co-exist in the Ara^–2^ population exhibited opposite trends: the Ara^–2S^ clone produced 74% of large OMVs while the Ara^–2L^ clone mostly produced small OMVs (69%). Calculation of the number of OMVs produced per cell in the 1 L culture finally revealed that all evolved clones produced less OMVs than the ancestor especially for the Ara^–5^ clone, which exhibited a ~10-fold reduction in OMVs production (Figure 1B and Figure S1C). These results clearly indicate that OMV production by *E. coli* has similarly evolved towards the release of two types of OMVs in three independent populations (and four distinct lineages) but with peculiarities regarding size and abundance distribution that may depend on the precise evolutionary history of each LTEE population.

### 2.3. OMVs Biochemical Features Have Changed over 50,000 Generations of E. coli Evolution

**Figure 2:**
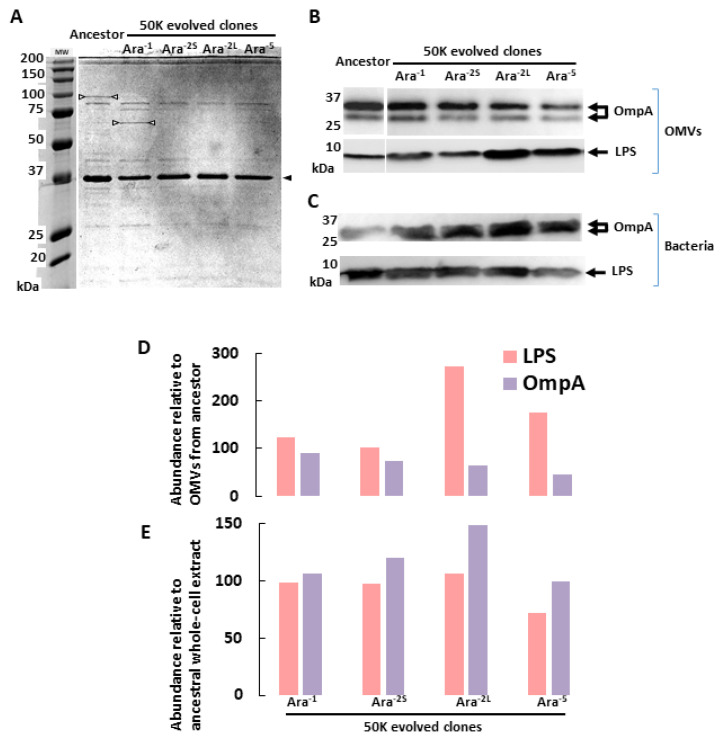
*E. coli* adaptation to the LTEE conditions leads to biochemical changes in OMV production. (**A**) Protein profiles of OMV preparations: equivalent amounts of OMVs (7.5 µg of total lipids) were separated on a 12% SDS-PAGE and revealed by Coomassie blue staining. The black arrowhead points to the major OmpA band migrating at the expected apparent molecular weight of 35 kDa. The white arrowheads point to unknown proteins that vary in abundance depending on the analyzed OMV preparation. (**B**) Equivalent quantities from each OMV preparation (corresponding to 7.5 µg of lipids) were separated on a 12% SDS-PAGE, transferred to nitrocellulose, and revealed by immunoblot using either polyclonal anti-OmpA or monoclonal anti-*E. coli* LPS antibodies. (**C**) Whole cell extracts of each bacterial clone (corresponding to 3 µg of proteins) were separated on a 12% SDS-PAGE, and immunoblotted using either polyclonal anti-OmpA or monoclonal anti-*E. coli* LPS antibodies. (**D**,**E**) The OmpA and LPS signals from immunoblots illustrated in B and C were quantified using Imagelab and presented as percentages relative to the ancestral strain signals for OMV preparations in D or for whole cell extracts in E.

To further characterize the evolution of OMVs’ production in the four LTEE 50K-evolved clones, we compared their respective protein profiles to that of OMVs produced by the ancestor (Figure 2A). Equivalent amounts of OMVs (corresponding to 7.5 µg of total lipids) from each preparation were analyzed by SDS-PAGE. For all OMV fractions, the Coomassie stained profiles contained one very abundant protein band at 35 kDa that corresponds to OmpA [24], the major porin in the outer-membrane of *E. coli* (black arrows on Figure 2A). Nevertheless, low abundance protein bands differed when comparing OMV protein profiles of ancestral and evolved clones (white arrows on Figure 2A). Accordingly, it suggests that bacterial adaptation to the LTEE conditions may either have impacted expression of proteins that were embedded in the OMVs from the ancestral strain or have modified the protein sorting process during OMV biogenesis in evolved strains. The OmpA proteins in the different OMVs preparations were then quantified by immunoblot using specific antibodies (Figure 2B). The signal intensity of each detected band was quantified and expressed as a percentage of the signal intensity measured for OMVs produced by the ancestral strain (Figure 2D). The OmpA signal was lower in the OMVs from all the evolved clones, especially for the Ara^–2L^ and Ara^–5^ clones where it corresponded to 60% and 50% of the signal from the ancestral strain, respectively. We confirmed that these differences did not result from down-regulation of OmpA expression in the evolved clones since immuno-blots of whole bacterial cell extracts indicated that OmpA levels tended to be higher in 50K-evolved clones when compared to the ancestral strain (Figure 2C,E). Because Lipopolysaccharide (LPS) molecules are commonly found in the membrane of OMVs, we next investigated whether the LPS content of OMVs also varied when comparing the OMVs from the evolved clones to those from the ancestral strain by immunoblot with anti-LPS specific antibodies (Figure 2B,D). The amount of LPS was higher in OMVs produced by 50K-evolved clones with up to two and three-fold increase for Ara^–2L^ and Ara^–5^ clones, respectively. Again, this increased signal was not correlated to an increase of LPS content in whole bacterial cell extracts (Figure 2C,E), hence suggesting that bacterial evolution through 50,000 generations in the LTEE conditions resulted in modulation of the biochemical features of the OMVs produced by *E. coli.*

### 2.4. OMVs from the Ancestral Strain Are Capable of Fusion with Bacterial Cells of the Ancestor and of 50K Evolved Clones

**Figure 3:**
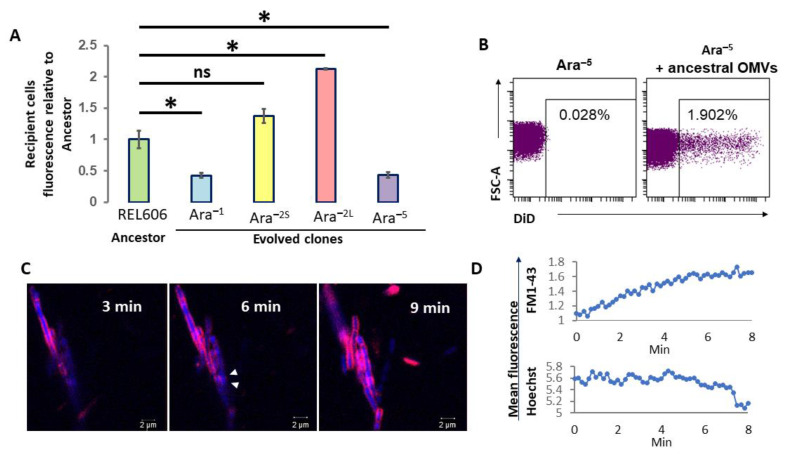
OMVs produced from the ancestral strain fuse with bacterial cells of the ancestral clone and 50K evolved clones. (**A**) Spectroscopic analysis. DiL-labelled OMVs from the ancestral strain were incubated with whole-cell suspension of the ancestral strain (REL606) and each of the 50K-evolved clones (Ara^–1^, REL11330; Ara^–2S^, REL11335; Ara^–2L^, REL11333; Ara^–5^, REL11339). Fluorescence levels acquired by each type of recipient bacteria were measured after 30 min of incubation, at 37 °C, and expressed relative to that measured for ancestral recipient bacteria. The experiment was performed 3 times, *: *p* < 0.05; ns: not significant. (**B**) Cytometric analysis. Cells of the Ara^−5^ evolved clone were incubated with non-labelled (left panel) or DiD-labelled (right panel) OMVs from the ancestral strain and the frequency of labelled recipient cells was quantified by flow cytometry. The y axis (FSC: Forward Scatter) corresponds to the diameter of the detected bacterial cells, while the x axis corresponds to the intensity of the DiD fluorescence. Cells were gated to avoid bacterial auto-fluorescence and to detect DiD fluorescence only. (**C**) Live confocal microscopic analysis. FM1-43-labelled OMVs from the ancestral strain (red) were incubated at room temperature with bacterial cells of the Ara^−5^ evolved clone pre-labelled with Hoechst 33,342 (blue). White triangles indicate 2 FM1-43-labelled OMVs fusing with the same recipient bacterial cell. The 3 images were taken from a live 9-min sequence at 3, 6 and 9 min after addition of fluorescently labelled OMVs. This sequence is representative of 2 experiments performed with FM1-43 and one with DiL markers, all providing similar results. Some bacteria remained blue and did not fuse with OMVs. (**D**) 8 min-follow-up of the mean FM1-43 and Hoechst 33342 fluorescence intensities for one particular bacterial cell, as quantified by the ImageJ software using images similar to that presented in C.

To investigate the putative role of OMVs in cell–cell communication in LTEE populations, we next assessed the capacity of OMVs from the ancestral strain to fuse with *E. coli* bacterial cells. First, the OMVs prepared from a mid-log phase culture of the ancestral strain were fluorescently labelled with DiL for spectrometric or microscopy analyses, or with DiD for flow cytometry. To ensure that the fluorescent dyes could not directly label whole bacterial cells, the fluorescent vesicles were washed by PBS to remove free dyes before to be added to suspension of non-fluorescent whole cells. We then incubated fluorescent OMVs devoid of free dyes with bacterial recipient cells corresponding to either ancestral cells or cells from each of the four 50K-evolved clones for 30 min. Whole bacterial cells were then separated from free OMVs by low-speed centrifugation and the fluorescence of the whole bacterial cell pellets was measured by spectrophotometry. The fluorescence level measured for evolved recipient cells was expressed relatively to that measured for ancestral recipient cells having fused with the fluorescently labelled OMVs they produced which was set to 1 (Figure 3A). For both the Ara^−1^ and Ara^−5^ 50K-clones, the fluorescence levels of recipient cells were only 46% of that measured for the ancestor indicating that those bacterial cells were less prone to fuse with OMVs from the ancestral strain. By contrast, cells from the Ara^–2S^ clone fused with OMVs from the ancestral strain as efficiently as the ancestral cells, as attested by the mean relative value of 1.4 which was not statistically different from 1 (Figure 3A). Finally, recipient cells from the Ara^–2L^ clone exhibited a significant 2.1-fold increase in fluorescence level indicating a higher capacity to fuse with OMVs from the ancestral strain.

Having demonstrated that OMVs from the ancestral strain fuse in various proportions with bacteria they originate from, as well as with cells from all 50K-evolved clones, we next chose to analyze their fusion with Ara^–5^ evolved cells in more details using flow cytometry and confocal microscopy. Flow cytometry showed that when evolved cells were mixed with non-labelled OMVs from the ancestral strain, as few as 0.03% of the cells became fluorescent (Figure 3B, left panel), while this proportion reached 1.9% when evolved cells were previously mixed with DiD-labelled OMVs from the ancestral strain (Figure 3B, right panel). It was thus clearly demonstrated that OMVs from the ancestor strain do fuse with evolved bacteria, although with low efficiency in the case of the Ara^–5^ clone.

Using live confocal microscopy, we finally visualized at room temperature the fusion process between FM1-43-labelled OMVs from the ancestral strain and Hoechst 33342-labelled evolved bacterial cells. The resolution of confocal microscopy did not allow tracking individual OMVs. However, as shown in Figure 3C, the OMV FM1-43 signal spread quickly in the bacterial membrane with most of the bacterial surface being fluorescent after 9 min of incubation. While the Hoechst fluorescence intensity of the selected bacteria remained relatively steady for up to 5 min, it then gradually declined reflecting natural fluorescence quenching. Inversely FM1-43 fluorescence increased gradually, exhibiting a typical saturation curve (Figure 3D). This demonstrated that the observed variations in the fluorescence intensity were not due to bacteria drifts during the acquisition of images. We also noticed that the fluorescence emerged at the same time at multiple locations along one particular bacterium (white triangles at 6 min in Figure 3C) and then spread, hence indicating that multiple OMVs did fuse almost simultaneously with the same bacterial cell.

## 3. Discussion

The release of OMVs by Gram-negative bacteria has long been documented [25] but the mechanisms sustaining their biogenesis are not yet fully understood [4]. Here, we started to investigate the dynamics of OMVs production by *Escherichia coli* in evolving populations from the LTEE. We compared the biophysical and biochemical features of OMVs produced by the *E. coli* strain used as ancestor in this evolution experiment [11,13,26] to those produced by its descendants after 50,000 generations of adaptation to laboratory conditions. We showed that bacterial adaptation to the LTEE conditions is accompanied by profound rewiring of the OMV production process with both the size and the LPS and protein contents of the 50K-evolved clones’ OMVs differing from those of the ancestral strain. Moreover, we showed that OMVs from the ancestral clone of *E. coli* do fuse with bacterial cells producing them but also with cells of all the tested LTEE 50K-evolved clones. Altogether, these results question the role of OMVs in long-term adaptation of bacterial populations to new environmental conditions including bacteria–host interactions (see companion paper).

OMVs were prepared from the ancestral strain and evolved clones, all cultured in the same Davis medium as that used for the propagation of the LTEE populations, yet with a 40 times higher glucose concentration in order to reach sufficient cell densities. The cultures were stopped at their mid-log phase of growth since bacteria have been shown to produce more OMVs during this culture phase [3]. It is indeed during this culture phase that bacteria actively divide and that bacterial cell death is limited, thus preventing too many extracellular vesicles that would result from cell lysis. To optimize the number of collected OMVs from culture supernatants while minimizing unwanted OMV fusions, we used a one hour ultracentrifugation at 150,000× *g* rather than the commonly used protocol with longer ultracentrifugation time but at reduced speed (18 h at 80,000× *g*) [5]. This optimized protocol led us to calculate that ~170 OMVs were produced per bacterial cell of the *E. coli* ancestral strain (Figure 1B). This number appears larger than that previously determined using electronic microscopy [27]. Nevertheless, the methodology we used implies that the number of collected OMVs does not correspond to what one cell can produce at a given time as observed using microscopy but rather to the total amount of OMVs produced by the bacterial population during the entire culture.

Comparing the biophysical features of 50K-evolved clones’ OMVs with those from their common ancestor, we showed parallel evolution for two distinct traits: first, all the tested clones evolved to produce two types of OMVs, small ones similar in radius to those produced by the ancestral strain but also larger ones with a 2.5 to 4.5 increase in radius length (Figure 1A); second, all tested clones have evolved to produce a lower amount of OMVs with a 4.5 to 10.5-fold reduction in the number of OMVs produced per cell (Figure 1B). Because these parallel changes repeatedly evolved in four 50K-evolved clones isolated from three independent populations, it suggests that these changes (i) may be advantageous per se and are hence promoted by natural selection during bacterial adaptation to the LTEE conditions, or (ii) that they are the indirect consequences of other phenotypic trait changes selected during evolution. Among the latter is the evolution of the cell volume which was shown to have repeatedly evolved towards a larger cell volume in all 12 LTEE populations after 50,000 generations [28]. On the contrary, the mutator phenotype that evolved in a majority of the 12 LTEE populations [14] did not appear to cause changes in OMV production since the mutators (Ara^–1^, Ara^–2S^ and Ara^–2L^) and the non-mutator (Ara^–5^) clones tested in the present study exhibited similar trends regarding their OMV production (Figure 1A). Besides these parallel changes, we also showed that the four 50K-evolved clones differed in their OMV production. Although they all produced both small and large OMVs, each clone exhibited peculiarities regarding the radius and the proportion of large OMVs it produced: both the Ara^–1^ and Ara^–2S^ clones evolved to produce a majority (66 and 74%, respectively) of large OMVs with similar average radii (50 and 55 nm, respectively); the Ara^–2L^ clone produced a low amount (31%) of very large OMVs with an average radius of 89 nm; the Ara^–5^ clone produced almost exclusively (96%) large OMVs (64 nm radius). These differences most likely relied on some subtle evolutionary traits that distinguish the various evolved clones, including the repertoire of selected mutations they harbor [14]. In this regard, it is interesting to note that an adaptive mutation in *pbpA*—the product of which catalyzes the final step of peptidoglycan synthesis—was previously shown to be selected early in the Ara^–5^ population and to contribute to cell volume increase in this population [18]. Considering the known role of the peptidoglycan in OMV biogenesis [29], this mutation could lead the Ara^–5^ evolved clone to produce almost exclusively large OMVs. Another interesting point concerns the differences between the two clones isolated from the Ara^–2^ population. In this population, two ecotypes diverged from the common ancestor at ~6K generations and established long-term co-existence involving niche construction and cross-feeding interaction. The L ecotype (L stands for “Large” owing to its large cell-size and colonies diameter on solid medium) grows faster on glucose but secretes acetate that the S ecotype (S stands for “Small” vs. “Large”) better exploits [23]. The two Ara^–2^ -50K-evolved clones investigated here strongly differed in their OMV production with the S ecotype clone (Ara^–2S^) producing a majority of large OMVs (50 nm radius) while the L ecotype clone (Ara^–2L^) mainly produced small OMVs (17 nm radius) (Figure 1A). This difference is consistent with the previously characterized phenotypic divergence between these two ecotypes after they started to co-exist [30] but may also constitute a not-yet-investigated parameter of their long-term interaction.

Comparing biochemical features of 50K-evolved clones’ OMVs with those of their ancestor, we showed that they differ in both their protein and LPS contents. The OMV protein profiles from both the ancestral and the evolved strains exhibited few protein bands. In all OMV protein profiles, the major band corresponded to an apparent molecular weight of ~35 kDa, typical of the major outer-membrane porin, OmpA in *E. coli* (Figure 2A). Nevertheless, comparison of the protein profiles of the OMVs produced by the ancestral versus evolved strains showed different banding patterns, suggesting that bacterial adaptation to the LTEE conditions may have changed the protein-sorting mechanisms associated with the biogenesis of OMVs as specific cargo [28]. Accordingly, the level of the OmpA protein, known to contribute to membrane stability and bacterial resistance to environmental stress [29], was shown to be lower in OMVs from the evolved clones (Figure 2B,D) independently of any OmpA down-regulation in evolved cells (Figure 2C,E). Similarly, OMVs from the evolved clones were shown to contain more LPS (Figure 2B,D) independently of any LPS enrichment in the whole-cell extracts of the evolved clone (Figure 2C,E). Hence, these results highlight that bacterial adaptation to the LTEE conditions led to changes in the process of OMV biogenesis, the causes of which could be identified based on phenotypic and genotypic properties that are known to differentiate the ancestral strain from the evolved clones.

Using spectroscopy, we showed that the OMVs from the ancestral strain do fuse with the bacterial cells producing them but also with cells of all tested 50K-evolved clones, albeit with variable efficiency (Figure 3A): both the Ara^−1^ and Ara^−5^ 50K-evolved clones were ~50% less efficient in incorporating OMVs from the ancestral stain as compared to the ancestral strain while the two Ara^−2S^ and Ara^−2L^ clones exhibited a 1.5- and 2-fold enhanced fusion capacity, respectively. This difference in fusion capabilities likely results from variable lipid and protein composition of both the OMV and bacterial membranes, which will be important to investigate. Although we demonstrated that the OMVs from the ancestral strain do fuse with 50K-evolved clones, we nevertheless showed that fusion was a rare event with less than 2% of the Ara^−5^ 50K-evolved bacterial cells incorporating the fluorescence from the OMVs from ancestral stain after 30 min of incubation (Figure 3B). Nevertheless, using live confocal microscopy to track fusion between fluorescently labelled OMVs from the ancestral strain and cells from the Ara^−5^ 50K-evolved clones, we showed that fusion is a rapid process that starts within the first minutes of contact and that several OMVs can fuse with the same bacterial cell (Figure 3C,D). Vesicle fusion with recipient bacteria was already observed by the horizontal gene transfer of antibiotic resistance genes [31]. Here, we visualized directly these fusions using fluorescently labelled OMVs.

## 4. Materials and Methods

### 4.1. Bacterial Growth and Production of OMVs

Bacterial cells were revived overnight from glycerol stocks stored at −80 °C by starting a pre-culture at 37 °C, 180 rpm, in 10 mL of DM1000 (Davis medium: 30.6 mM K_2_HPO_4_; 15 mM KH_2_PO_4_; 6.2 mM (NH_2_)_4_SO_4_; 2 mM Na_3_C_6_H_5_O_7_; 0.83 mM MgSO_4_; 7.54 µM thiamine, supplemented with 5.55 mM (or 1000 mg/L) glucose (Glc)). A total of 10 mL of the revived bacteria were transferred into a 5 L Erlenmeyer glass-flask containing 1 L of DM1000. The flask was placed at 37 °C, under agitation at 180 rpm until the culture reached the OD_450_ (measured using an Eppendorf Biospectrometer, Hamburg, Germany) corresponding to the selected strain’s mid-log phase (Table 1). A 1 mL aliquot of the bacterial culture was kept at 4 °C for CFU determination. The rest of the culture was used to collect OMVs with intermediate recovery and specificity as defined by the Minimal Information for Studies of Extracellular Vesicles (MISEV) guidelines [32]. The cultures were transferred to 500 mL centrifugation bottles and bacterial cells were pelleted by a 30 min centrifugation at 5000× *g*, 21 °C, in a Beckman Coulter Avanti J-E JSE02C09 centrifuge (Brea, United States). The resulting ~1 L supernatant was filtered using a 0.22 µm filter (steritop Millipore, polyethersulfone membrane Burlington, United States) to eliminate any contaminating cells and transferred into a clean bottle. The OMVs from this sterile 1 L solution were finally pelleted by ultracentrifugation for 1 h at 150,000× *g* (41,000 rpm), 4 °C, using a Beckman Coulter Optima XE 90 ultracentrifuge (Type 45 Ti Fixed-Angle Titanium Rotor, k-Factor = 133, Polycarbonate tubes, Brea, United States) and no brake to prevent the dispersion of the OMV pellets. According to the theoretical framework described in [33], these ultracentrifugation parameters ensure full sedimentation of vesicles with diameters ranging from 31 to 200 nm. The resulting OMV pellets were ultimately dispersed into 1 mL of 0.22 µm-filtrated phosphate buffered saline (PBS) solution (137 mM NaCl, 2.7 mM KCl, 10 mM Na_2_HPO_4_, 1.8 mM KH_2_PO_4_) in polypropylene tubes and stored exclusively at 4 °C until further use to limit cryopreservation artefacts.

### 4.2. Determination of the OMV Size Distribution by Dynamic Light Scattering (DLS)

Dynamic Light Scattering allows the determination of nanoparticles’ size distribution in a complex mixture (radii within the range of 0.1 to 5000 nm). A total of 15 µL of each OMVs preparation were loaded in a Wyatt Dynastar instrument (Santa Barbara, United States) and 10 DLS acquisitions of 5 s each, repeated 3 times, were performed at a constant temperature of 25 °C. The obtained size distribution was used to calculate the weighted average radius of OMVs considering one (ancestor’s OMVs) or two (evolved clones OMVs) peaks as well as the repartition of the OMVs in the 2 size categories, if any.

### 4.3. Quantification of the Proteins Present in the OMVs

A bovine serum albumin (BSA) solution at 1 mg/mL in PBS was used to prepare a protein standard curve ranging from 0 to 650 µg/mL. A total 10 µL of OMVs’ suspension was mixed with 2 µL of 1.5% Triton X100 (Sigma, St. Louis, MI, United States) and 13 µL of PBS. The mix was vortexed and incubated at room temperature for 5 min in order to solubilize the OMVs’ membrane. A mix constituted of 50 parts of bicinchoninic acid (BCA) (Sigma) and 1 part of copper II sulfate (Sigma) was prepared extemporaneously, and 200 µL of this mix were added to each well of a transparent, flat-bottom 96-well plate (Corning, Glendale, CA, United States). A total of 25 µL of a BSA standard solution or the OMV preparations were added in wells containing the BCA/Copper II mix. After incubation at 37 °C for 30 min to allow the development of the purple color, the absorbance of each well was measured at 536 nm using a Varioskan spectrophotometer (Thermofisher, Waltham, MA, United States). All the standard and sample solutions were prepared in triplicates.

### 4.4. Quantification of the Lipids Present in OMVs 

The lipids present in OMVs’ preparations were quantified using a fluorescence assay based on the incorporation of a fluorescent lipophilic molecule (1-Anilinonaphthalene-8-Sulfonic Acid, ANS, Sigma) in their membrane. Artificial liposomes prepared from a mix of lipids representing the most abundant lipids of bacterial membranes were used as reference to obtain a lipid standard curve. A 16.6 mg/mL lipid stock solution was prepared by mixing 160 µL of 36 mM (25 mg/mL) phosphatidyl ethanolamine (PE; Avanti, Alabaster, AL, USA), 160 µL of 36 mM (25 mg/mL) phosphatidyl glycerol (PG) and 32 µL of 14 mM (10 mg/mL) cardiolipin (CL) in a glass tube. After drying the lipid film under a N_2_ stream, lipids were dispersed in 500 µL of a [50 mM Tris, 200 mM KCl, pH 7.5] solution leading to spontaneous formation of multi-lamellar vesicles. This solution was frozen in liquid N_2_, then heated 4 times at 50 °C, before being sieved 5 times through an extruder (Avanti) equipped with a 0.2 µm-diameter pore filter and 19 times through the same extruder equipped with a 0.1 µm-diameter pore filter (Avanti).

The ANS assay working solution was prepared by mixing 20 µL of 0.3% ANS (in ethanol) with 6 mL of [50 mM Tris, 200 mM KCl, pH 7.5] solution. A total of 200 µL of this ANS working solution was distributed into each well of a black, flat-bottom 96-well plate (Corning), to which 1, 2 or 3 µL of a 1 mg/mL liposome solution, or 1 µL of the OMVs’ preparation were added. Each vesicle sample (liposomes or OMVs) was triplicated. The fluorescence was measured using a Varioskan fluorimeter (Thermofisher) after setting up the excitation wavelength at 350 nm and the emission wavelength at 512 nm (100 ms time reading frame).

### 4.5. Estimation of the Number of OMVs Produced per Bacterial Cell

The number of CFUs was determined using the correspondence of OD_450_ = 1 to 4 × 10^8^ bacteria/mL. To determine the number of OMVs in each preparation, we first quantified their lipid contents using the ANS method and a standard curve obtained with artificial liposomes (Supplementary Figure S1B). Based on this quantity in gram of lipids (q) and an average lipid molecular weight of 700 g/mole, we calculated the total number of lipid molecules in the preparation as n = (q/700) × N, with N being the Avogadro number. Assuming that OMVs are perfect spheres made of 2 lipid leaflets and considering the average phospholipid polar head surface as 0.65 nm^2^ (see reference [34]), the total number of lipid molecules in one OMV of a radius *r* could be calculated as (2 × 4π*r*^2^)/0.65. Accordingly, we ultimately determined the number of OMVs in a preparation as n × 0.65/2 × 4π*r*^2^. If both small and large OMVs are present in the same preparation, we considered that the total amount of lipids (n) is distributed according to the abundance of each type of OMVs as determined by DLS analysis. Finally, the number of OMVs produced per bacterial cell was estimated by dividing this number of OMVs by the number of CFUs determined for the corresponding culture.

### 4.6. Analysis of the OMV Proteins by SDS-PAGE and Coomassie Blue Staining

OMV preparations corresponding to 7.5 µg of each type of OMV lipids were mixed with 4 µL of 4X Laemmli sample buffer (Bio-Rad, Hercules, United States). The mix was denatured at 95 °C for 10 min, loaded into the wells of a 12% polyacrylamide gel calibrated with 5 µL of Precision Plus Protein™ All Blue Prestained Protein Standard molecular weight marker (Bio-Rad). The proteins were separated for 1 h 30 min at 30 mA, 100 V. The gel was stained in Coomassie blue R solution (Sigma, St. Louis, United States) for 30 min, de-stained in several baths of de-staining solution [10% (*v*/*v*) acetic acid, 70% (*v*/*v*) ethanol, 20% (*v*/*v*) H_2_O] and photographed using a ChemiDoc apparatus (Bio-Rad). 

### 4.7. Immunoblots

OMV solutions corresponding to 7.5 µg of each type of OMV lipids were mixed with 4 µL of 4X Laemmli sample buffer (Bio-Rad). Whole cell extracts of each bacterial clone (corresponding to 3 µg of proteins) were mixed with 4 µL of 4X Laemmli sample buffer (Bio-Rad). Following SDS-PAGE, the proteins and associated LPS were transferred from the polyacrylamide gel to a nitrocellulose membrane (Bio-Rad) using a Trans-Blot turbo apparatus (Bio-Rad) according to the manufacturer’s recommendations. The membrane was blocked for 30 min in 5% non-fat dry milk in TBS-T [50 mM Tris, 200 mM NaCl, pH 7.5, 0.1% Tween-20], then incubated overnight in 2.5% non-fat dry milk in TBS-T containing either mouse monoclonal anti *E. coli* LPS IgGs (clone 2D7/1 (ab35654), Abcam, Cambridge, United Kingdom) diluted 1/2000 or rabbit anti-OmpA polyclonal antibodies (Epigentek, Farmingdale, United States) diluted 1/2000. After two washes of 10 min each in TBS-T, the membranes were incubated for 3 h, respectively, in 2.5% non-fat dry milk in TBS-T containing anti-mouse polyclonal antibodies coupled to horse radish peroxidase [HRP] and diluted 1/10,000 or containing HRP-conjugated anti-rabbit IgG F(ab’)_2_ fragment (Sigma) diluted 1/10,000. After washes in TBS-T, the membranes were revealed by ECL using a Clarity Western substrate kit (Bio-Rad) and photographed using a ChemiDoc apparatus (Bio-Rad).

### 4.8. OMV Fusion Experiments with Bacterial Cells

The prepared OMVs were fluorescently labelled using different dyes depending on the subsequent analysis: DiL (1,1′-Dioctadecyl-3,3,3′,3′-Tetramethylindocarbocyanine Perchlorate, DiIC18(3), Thermofisher) for spectroscopy; DiD (1,1′-Dioctadecyl-3,3,3′,3′-Tetramethylindodicarbocyanine Perchlorate, DiIC18(5), Thermofisher) for cytometry; and FM1-43 (N-(3-Triethylammoniumpropyl)-4-(4-(Dibutylamino) Styryl) Pyridinium Dibromide, Invitrogen, Waltham, United States) for microscopy. All the reaction components were pre-heated for 10 min at 37 °C before use to ensure solubilization of the dyes that are supplied in dimethyl sulfoxide. In all cases, 50 µL of OMVs preparation at 1.5 mg/mL in lipids were mixed with 1 µL of the dye in 1 mL PBS. The mix was homogenized for 1 h at 37 °C, 350 rpm, in a thermomixer (Eppendorf, Hamburg, Germany), then centrifuged for 3 h at 21,000× *g*, 21 °C. The pellet of labelled OMVs was dispersed in 60 µL of PBS and kept at 37 °C in the dark until being used.

For spectroscopic analyses, the selected bacteria were grown in 1 mL of DM1000 until the culture reached an OD_450_ = 1. After a 5 min centrifugation at 5000× *g*, the supernatant was discarded and the bacterial pellet dispersed in 1 mL of PBS. A total of 10 µL of the DiL-fluorescently labelled OMVs (10^10^) were added to 200 µL of the resulting bacterial suspension and the mix was incubated for 30 min, at 37 °C, 350 rpm, in a thermomixer. Bacteria were then recovered by low-speed centrifugation preventing OMVs sedimentation (5 min, 5000× *g*) and the cell pellet was dispersed in 200 µL of PBS. The suspension was transferred to a well of a flat-bottom 96-well black plate (Corning) and DiI fluorescence was measured using a Varioskan fluorimeter (Thermofisher) (excitation = 550 nm, emission = 600 nm, 100 ms time reading frame).

For flow cytometry analyses, we used the same OMVs labelling protocol as for spectroscopic analyses but using the DiD fluorescent dye. We incubated 10^6^ unlabeled bacterial cells dispersed in 1 mL with 10^10^ fluorescently labelled OMVs (i.e., 10,000 OMVs per recipient bacterial cell) in 1.5 mL Eppendorf tubes for 30 min. The free OMVs were then removed by one cycle of low-speed centrifugation (5 min, 5000× *g*) and the cell pellets were dispersed in PBS. The washed bacterial pellets were ultimately dispersed in 1 mL PBS and their fluorescence analyzed using a FACS Canto II (BD Biosciences, Le Pont de Claix, France) flow cytometer. The analysis was performed on singlet cells defined by FSC-A/FSC-H (forward scatter-amplitude and -height) and SSC-A/SSC-W (side scatter-amplitude and -height). Then the bacteria were gated on FSC-A/SSC-A to determine the percentage of labelled bacterial cells based on their acquisition of DiD-fluorescence.

For confocal analyses, bacteria were grown in 1 mL of DM1000 up to OD_450_ = 1, pelleted by centrifugation, and dispersed in 1 mL of PBS. Bacterial nucleoids were first labelled for 10 min with 0.1% Hoechst 33342 (Sigma). The fusion between FM1-43-labelled OMVs and Hoechst 33342-labelled bacteria was performed directly on a glass slide by mixing 2 µL of the Hoechst-labelled bacterial preparation, 1 µL of FM1-43-labelled OMVs (10^9^) (1 mg/mL lipid concentration), and 2 µL of Vector shield (Sigma-Aldrich, St. Louis, United States). Preparations were observed using a Zeiss LSM 70 Axio Observer confocal microscope (Carl Zeiss, Jena, Germany) equipped with a Plan-Apochromat 63×/1.40 Oil DICM27 objective and a 4.5 zoom. Hoechst was excited with a 405 nm laser diode and emission collected through a 430–490 nm band-pass filter. FM1-43 was excited with a 543 nm HeNe laser and emission collected through a 590 nm long-pass filter. The optical resolution was approximately 0.2 µm/pixel. The resulting images were analyzed using the ImageJ software (v1.48, https://imagej.nih.gov/ij/) with the Bio-format plugin.

### 4.9. Statistical Analyses

Lipid and protein quantifications as well as fusion experiments (spectrometry) were performed in triplicates. The significance of the results was determined using GraphPad Prism 5 by performing a Student’s *t*-test with a confidence interval of 95%, which means that the results were significant when the *p*-value was lower than 0.05.

## 5. Conclusions

In this study we have started to characterize the dynamics of OMVs production by *E. coli* in evolving populations from the LTEE. We showed that OMVs biogenesis was profoundly rewired after 50,000 generations of adaptation to glucose-limited minimal medium. Because the evolutionary history of the LTEE populations has been extensively characterized both at the genotypic and phenotypic levels [11,13,20], future work will allow the identification of the causes of these changes in OMVs production, hence providing the opportunity to reveal new determinants controlling OMV production in *E. coli*. Moreover, we showed that the propensity of *E. coli* OMVs to fuse with whole bacterial cells depends on the recipient cells, hence questioning the mechanisms responsible for these differences that could be investigated using various clones isolated from LTEE populations.

## Figures and Tables

**Table 1:** LTEE bacterial clones used in this study. The table indicates the population they originated from, the evolutionary time they were isolated and the time at which pure cultures in DM1000 were stopped before OMVs isolation with the corresponding mid-log growth phase optical density (OD) at 450 nm, and their bacterial titer in colony-forming units per milliliter.

Population Clones	Number of Generations	Time Length of Culture	^OD^_450_ at Mid-Log Growth Phase	Bacterial Titer (CFUs/mL)
Ara^−^ ancestor clone REL606	0	5 h 50 min	1	3.89 × 10^8^
Ara^−1^ clone REL11330	50,000	3 h 30 min	1.5	1.5 × 10^8^
Ara^−2S^ clone REL11335	50,000	4 h 30 min	1.4	2.38 × 10^8^
Ara^−2L^ clone REL11333	50,000	3 h 15 min	1.4	2.38 × 10^8^
Ara^−5^ clone REL11339	50,000	4 h 15 min	1.6	2.7 × 10^8^

## Data Availability

Not applicable.

## Supplementary Materials

The following data are available online at https://www.mdpi.com/article/10.3390/ijms232314580/s1.

## References

[1] Stentz R., Carvalho A.L., Jones E.J., Carding S.R. (2018). Fantastic Voyage: The Journey of Intestinal Microbiota-Derived Microvesicles through the Body. Biochem. Soc. Trans..

[2] He B., Hamby R., Jin H. (2021). Plant Extracellular Vesicles: Trojan Horses of Cross-Kingdom Warfare. FASEB BioAdvances.

[3] Gill S., Catchpole R., Forterre P. (2019). Extracellular Membrane Vesicles in the Three Domains of Life and Beyond. FEMS Microbiol. Rev..

[4] Nagakubo T., Nomura N., Toyofuku M. (2020). Cracking Open Bacterial Membrane Vesicles. Front. Microbiol..

[5] Schwechheimer C., Kuehn M.J. (2015). Outer-Membrane Vesicles from Gram-Negative Bacteria: Biogenesis and Functions. Nat. Rev. Microbiol..

[6] Bittel M., Reichert P., Sarfati I., Dressel A., Leikam S., Uderhardt S., Stolzer I., Phu T.A., Ng M., Vu N.K. (2021). Visualizing Transfer of Microbial Biomolecules by Outer Membrane Vesicles in Microbe-Host-Communication in Vivo. J. Extracell. Vesicles.

[7] Chronopoulos A., Kalluri R. (2020). Emerging Role of Bacterial Extracellular Vesicles in Cancer. Oncogene.

[8] Yáñez-Mó M., Siljander P.R.M., Andreu Z., Zavec A.B., Borràs F.E., Buzas E.I., Buzas K., Casal E., Cappello F., Carvalho J. (2015). Biological Properties of Extracellular Vesicles and Their Physiological Functions. J. Extracell. Vesicles.

[9] Sartorio M.G., Pardue E.J., Feldman M.F., Haurat M.F. (2021). Bacterial Outer Membrane Vesicles: From Discovery to Applications. Annu. Rev. Microbiol..

[10] Kulp A., Kuehn M.J. (2010). Biological Functions and Biogenesis of Secreted Bacterial Outer Membrane Vesicles. Annu. Rev. Microbiol..

[11] Lenski R.E. (2017). Experimental Evolution and the Dynamics of Adaptation and Genome Evolution in Microbial Populations. ISME J..

[12] Blount Z.D., Maddamsetti R., Grant N.A., Ahmed S.T., Jagdish T., Baxter J.A., Sommerfeld B.A., Tillman A., Moore J., Slonczewski J.L. (2020). Genomic and Phenotypic Evolution of Escherichia Coli in a Novel Citrate-Only Resource Environment. eLife.

[13] Hindré T., Knibbe C., Beslon G., Schneider D. (2012). New Insights into Bacterial Adaptation through in Vivo and in Silico Experimental Evolution. Nat. Rev. Microbiol..

[14] Lenski R.E., Rose M.R., Simpson S.C., Tadler S.C. (1991). The American Society of Naturalists Long-Term Experimental Evolution in Escherichia Coli. I. Adaptation and Divergence During. Gener. J. Am. Soc. Aging.

[15] Wiser M.J., Ribeck N., Lenski R.E. (2013). Long-Term Dynamics of Adaptation in Asexual Populations. Science.

[16] Lenski R.E., Travisano M. (1994). Dynamics of Adaptation and Diversification: A 10,000-Generation Experiment with Bacterial Populations. Proc. Natl. Acad. Sci. USA.

[17] Philippe N., Pelosi L., Lenski R.E., Schneider D. (2009). Evolution of Penicillin-Binding Protein 2 Concentration and Cell Shape during a Long-Term Experiment with Escherichia Coli. J. Bacteriol..

[18] Stanek M.T., Cooper T.F., Lenski R.E. (2009). Identification and Dynamics of a Beneficial Mutation in a Long-Term Evolution Experiment with Escherichia Coli. BMC Evolutionary Biology.

[19] Tenaillon O., Barrick J., Ribeck N., Deatherage D., Blanchard J., Dasgupta A., Wu G., Wielgoss S., Cruveiller S., Medigue C. (2016). Tempo and Mode of Genome Evolution in a 50,000-Generation Experiment. Nature.

[20] Wielgoss S., Barrick J.E., Tenaillon O., Wiser M.J., Dittmar W.J., Cruveiller S., Chane-Woon-Ming B., Médigue C., Lenski R.E., Schneider D. (2013). Mutation Rate Dynamics in a Bacterial Population Reflect Tension between Adaptation and Genetic Load. Proc. Natl. Acad. Sci. USA.

[21] Consuegra J., Gaffé J., Lenski R.E., Hindré T., Barrick J.E., Tenaillon O., Schneider D. (2021). Insertion-Sequence-Mediated Mutations Both Promote and Constrain Evolvability during a Long-Term Experiment with Bacteria. Nat. Commun..

[22] Rozen D.E., Philippe N., Arjan De Visser J., Lenski R.E., Schneider D. (2009). Death and Cannibalism in a Seasonal Environment Facilitate Bacterial Coexistence. Ecol. Lett..

[23] Großkopf T., Consuegra J., Gaffé J., Willison J.C., Lenski R.E., Soyer O.S., Schneider D. (2016). Metabolic Modelling in a Dynamic Evolutionary Framework Predicts Adaptive Diversification of Bacteria in a Long-Term Evolution Experiment. BMC Evol. Biol..

[24] Tulkens J., De Wever O., Hendrix A. (2020). Analyzing Bacterial Extracellular Vesicles in Human Body Fluids by Orthogonal Biophysical Separation and Biochemical Characterization. Nat. Protoc..

[25] Bishop D.G., Work E. (1965). An Extracellular Glycolipid Produced by Escherichia Coli Grown under Lysine-Limiting Conditions. Biochem. J..

[26] Good B.H., McDonald M.J., Barrick J.E., Lenski R.E., Desai M.M. (2017). The Dynamics of Molecular Evolution over 60,000 Generations. Nature.

[27] Ellis T.N., Leiman S.A., Kuehn M.J. (2010). Naturally Produced Outer Membrane Vesicles from Pseudomonas Aeruginosa Elicit a Potent Innate Immune Response via Combined Sensing of Both Lipopolysaccharide and Protein Components. Infect. Immun..

[28] Qing G., Gong N., Chen X., Chen J., Zhang H., Wang Y., Wang R., Zhang S., Zhang Z., Zhao X. (2019). Natural and Engineered Bacterial Outer Membrane Vesicles. Biophys. Rep..

[29] Avila-Calderón E.D., Ruiz-Palma M.D.S., Aguilera-Arreola M.G., Velázquez-Guadarrama N., Ruiz E.A., Gomez-Lunar Z., Witonsky S., Contreras-Rodríguez A. (2021). Outer Membrane Vesicles of Gram-Negative Bacteria: An Outlook on Biogenesis. Front. Microbiol..

[30] Le Gac M., Plucain J., Hindré T., Lenski R.E., Schneider D. (2012). Ecological and Evolutionary Dynamics of Coexisting Lineages during a Long-Term Experiment with Escherichia Coli. Proc. Natl. Acad. Sci. USA.

[31] Yaron S., Kolling G.L., Simon L., Matthews K.R. (2000). Vesicle-Mediated Transfer of Virulence Genes from Escherichia Coli O157:H7 to Other Enteric Bacteria. Appl. Environ. Microbiol..

[32] Théry C., Witwer K.W., Aikawa E., Alcaraz M.J., Anderson J.D., Andriantsitohaina R., Antoniou A., Arab T., Archer F., Atkin-Smith G.K. (2018). Minimal Information for Studies of Extracellular Vesicles 2018 (MISEV2018): A Position Statement of the International Society for Extracellular Vesicles and Update of the MISEV2014 Guidelines. J. Extracell. Vesicles.

[33] Livshits M.A., Livshts M.A., Khomyakova E., Evtushenko E.G., Lazarev V.N., Kulemin N.A., Semina S.E., Generozov E.V., Govorun V.M. (2015). Isolation of Exosomes by Differential Centrifugation: Theoretical Analysis of a Commonly Used Protocol. Sci. Rep..

[34] Takamori S., Holt M., Stenius K., Lemke E.A., Grønborg M., Riedel D., Urlaub H., Schenck S., Brügger B., Ringler P. (2006). Molecular Anatomy of a Trafficking Organelle. Cell.

