# Supplementary material for: Extracellular Vesicles from 50,000 Generation Clones of the Escherichia coli Long-Term Evolution Experiment
Source: Int J Mol Sci. 2022 Nov 23;23(23):14580. doi: 10.3390/ijms232314580 (PMC9737989; doi:10.3390/ijms232314580)
Supplement: Supplementary file 1 [file ijms-23-14580-s001.zip › ijms-2000586-Supplementary Materials.pdf]

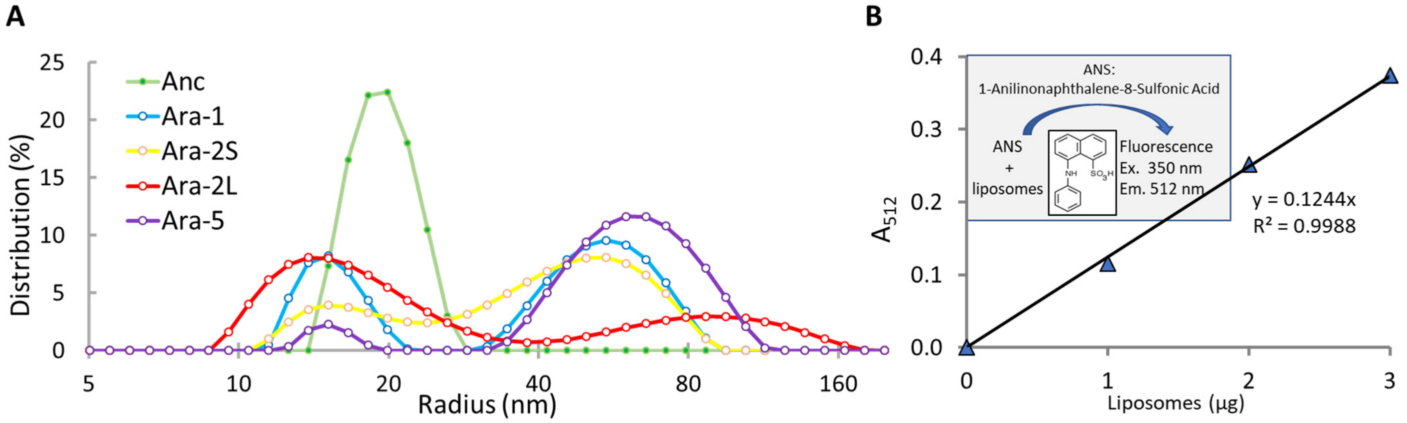

C

Vesicles	Ancestor	Ara^{-1} *		Ara^{-2S} *		Ara^{-2L} *		Ara^{-5} *	
		Small	Large	Small	Large	Small	Large	Small	Large
Weighted average radius (nm)	20	15	55	17	50	17	89	19	81
Min-Max radius (nm)	15.1-26.2	11.5-21.8	31.5-86.6	11.5-23.9	26.2-86.6	9.6-37.9	41.5-180.6	12.-18.2	34.6-114.1
Proportion	1	0.34	0.66	0.26	0.74	0.69	0.31	0.06	94
Total lipid (mg)	0.83 +/- 0.17	0.341		0.441		0.7		0.455	
Number of OMVs	$4.81x10^{13}$ +/- 0.96	$1.21x10^{12}$	$2.37x10^{12}$	$1.30x10^{12}$	$3.74x10^{12}$	$4.09x10^{12}$	$1.81x10^{12}$	$1.66x10^{11}$	$2.43x10^{12}$
Number of CFU	$2.87x10^{11}$ +/- 0.44	$9.6x10^{10}$		$1.90x10^{11}$		$1.90x10^{11}$		$1.62x10^{11}$	
Number OMV/cell	170.4 +/- 37.2	12.6	24.6	6.8	19.7	21.5	9.5	1.0	15.0

Figure S1: Biophysical characterization of **OMVs prepared from pure cultures of LTEE clones** (A) OMVs size distribution profiles as measured using Dynamic Light Scattering (DLS). (B) Principle and standard curve of the ANS assay used to quantify total lipid amount in OMVs preparations. This fluorescence assay is based on 1-Anilinonaphthalene-8-Sulfonic Acid (ANS) that behaves as a fluorochrome exclusively when bound to lipidic membranes. Ex.: excitation; Em.: emission. The standard curve used for the assay was obtained using various amounts of artificial liposomes built with a mix of Phosphatidyl Ethanolamine (PE)/Phosphatidyl Glycerol (PG)/Cardiolipin (CL) at a 40/40/20 molar ratio. (C) Summary of the data obtained from DLS and ANS assays that were used for calculation as explained in the material and methods section. The presented data are representative of 6 experiments for the ancestral strain (Anc, REL606) but only one for the 50K-evolved clones (*).
